# Age differences between sexual partners, behavioural and demographic correlates, and HIV infection on Likoma Island, Malawi

**DOI:** 10.1038/srep36121

**Published:** 2016-11-02

**Authors:** Roxanne Beauclair, Stéphane Helleringer, Niel Hens, Wim Delva

**Affiliations:** 1 International Centre for Reproductive Health, Ghent University, Gent, Belgium; 2 The South African Department of Science and Technology-National Research Foundation (DST-NRF) Centre of Excellence in Epidemiological Modelling and Analysis (SACEMA), Stellenbosch University, Stellenbosch, South Africa; 3 Bloomberg School of Public Health, Johns Hopkins University, Baltimore, USA; 4 Center for Statistics, Hasselt University, Hasselt, Belgium; 5 Centre for Health Economic Research and Modelling Infectious Diseases, Vaccine and Infectious Disease Institute, University of Antwerp, Wilrijk, Belgium; 6 Epidemiology and Social Medicine, University of Antwerp, Wilrijk, Belgium; 7 Rega Institute for Medical Research, KU Leuven, Leuven, Belgium

## Abstract

Patterns of age differences between sexual partners – “age-mixing” – may partially explain the magnitude of HIV epidemics in Sub-Saharan Africa. However, evidence of age-disparity as a risk factor for HIV remains mixed. We used data from a socio-centric study of sexual behaviour in Malawi to quantify the age-mixing pattern and to find associations between relationship characteristics and age differences for 1,922 participants. Three age difference measures were explored as predictors of prevalent HIV infection. We found that for each year increase in male participant age, the average age difference with their partners increased by 0.26 years, while among women it remained approximately constant around 5 years. Women in the study had larger within-individual variation in partner ages compared to men. Spousal partnerships and never using a condom during sex were associated with larger age differences in relationships of both men and women. Men who were more than five years younger than their partners had 5.39 times higher odds (95% CI: 0.93–31.24) of being HIV-infected than men 0–4 years older. The relationship between HIV-infection and age-asymmetry may be more complex than previously described. The role that women play in HIV transmission should not be under-estimated, particularly in populations with large within-individual variation in partner ages.

Age-mixing results from societal norms for how men and women of a population preferentially choose sexual partners from a certain age. It may impact the spread of HIV^1^,^2^,^3^ and partially explain the magnitude of HIV epidemics observed in Sub-Saharan Africa^4^. For instance, a modelling study, based on empirical data from Manicaland, Zimbabwe, implied that a relatively small decrease in the fraction of relationships where the male partner is older than the female partner might reduce the HIV prevalence among young women and their lifetime risk of HIV infection^5^. An ecological study of HIV risk factors in four countries – two with high and two with low HIV prevalence – found that women with older partners were more common in high HIV prevalence settings^6^.

The population-level effect of age-asymmetric relationships may be partially explained by HIV prevalence trends observed in several Sub-Saharan countries. In South Africa and Malawi, for example, HIV prevalence for men tends to peak in older age groups compared to women^7^,^8^,^9^, implying that if women are choosing partners from older age groups where the prevalence is higher than among men in their own age group, they are more likely to choose a partner who has HIV. Additionally, higher sexual risk behaviours have been associated with age-asymmetric relationships. Specifically, men in these relationships use condoms less frequently^10^,^11^, and have more concurrent partnerships^12^,^13^,^14^ than in relationships where men and women are of similar ages. Young women in age-asymmetric relationships also display behaviours and have characteristics which may put them at increased risk for HIV infection: increased reports of STI treatment or symptoms^10^,^11^,^15^, more lifetime partners^10^, earlier sexual debut^10^, and higher sex frequency^10^.

Observational studies, however, only provide some evidence of the impact of age-asymmetries in relationships on HIV risk. In Zimbabwe, increased age differences between partners raised a participant’s odds of being HIV positive^12^. Similarly, in a recent national survey in South Africa, HIV prevalence was higher in those who had age-disparate relationships – defined as a relationship where the partner is five or more years older than the participant – compared to those not in age-disparate relationships^7^. The positive association between age difference and HIV status may, however, only hold for younger women (e.g., less than 25 years old), whereas in older women who have partners older than themselves, HIV or STI risk declines^15^,^16^. On the other hand, an ecological study in Kenya found no association between the proportion of women who had an age-disparate relationship and HIV prevalence^17^. A study from a large population-based cohort in rural KwaZulu-Natal, South Africa also found no significant association between partner age differences and HIV incidence among women 15–30 years old^18^. Among women in this population who were 30 years or older, risk of HIV acquisition fell as age difference increased^19^.

These conflicting results may be explained by different geographical and cultural contexts. They may also be due to limitations of study designs and measurement of age-mixing. Most studies have focused on the association between age difference during the most recent partnership and HIV risk. Other aspects of age-mixing may also play a role. For example, the maximum age difference that an individual has had with his/her partner(s) may be an important determinant of HIV risk. HIV risk may also be associated with the variation in age differences across the partnerships of an individual^4^. Above and beyond the limitations to measurement and analysis of age-mixing, previous studies have neglected to examine the effects of men choosing older partners on HIV dynamics. In this paper, we used data from the Likoma Network Study in Malawi to test whether these understudied aspects of age-mixing are associated with HIV status.

## Methods

### Data Source

We used data from the Likoma Network Study (LNS), a sociocentric study of sexual networks and HIV on Likoma Island, Malawi that took place in 2006, 2007/08, and 2013. Data from the LNS were collected in several steps: first, a census of the island’s population was collected to obtain a list of potential members of the sexual networks; second, a sexual network survey was conducted with adult members of the population, during which respondents were asked to nominate their recent sexual partners; finally, these nominations were linked to the household census lists, in order to reconstruct maps of the sexual networks that connected inhabitants of the island.

The sexual network survey was administered using Audio Computer-Assisted Self-Interviewing (ACASI), and details of sexual partnerships were recorded for a maximum of five partners in the past three years. Participants were asked to name each of their partners in a recording headset and provide additional identifying information (e.g. location in the village, nicknames, etc.). A partner was defined as someone with whom the participant had had vaginal intercourse within the previous three years. For each partner, questions were asked about the location of their partner, frequency of sexual intercourse, condom use, and additional relationship characteristics.

While the LNS took place in different rounds, the study itself does not follow the strict definition of a cohort study. Some participants that were asked questions in 2006 did not participate in the 2007/2008 version of the study and vice versa. More importantly, some of the sexual behaviour and partner characteristic questions were different from round to round. For example, we do not have partner ages for those participating in 2006. Therefore, we used only data from the 2007/2008 version and treated it as a cross-sectional study. Details of the LNS participant flow have been outlined previously^20^. More details of the LNS study design, sampling, and data collection procedures have been published elsewhere^21^.

There were 2009 participants included in this study. However, 87 participants (4.3%) did not report any sexual partners and were thus excluded. Therefore, our secondary analysis includes the 1922 participants from the 2007/2008 round of the LNS that took part in the sexual behaviour survey and reported at least one partner in the past five years. Jointly they reported 3336 relationships, with the average number of partners per participant being 1.74 (Median: 1, IQR: 1–2).

### Measures

Each study participant self-reported his or her own age. The *age difference* in a given relationship was then defined as the male partner’s age minus the female partner’s age. Consequently, if the female partner in a relationship was older than her male partner, there would be a negative age difference. For each participant, we calculated the *mean* and *maximum age difference* between an individual and his/her partners. If a participant only reported one relationship then the *mean* and *max age difference* were the same. The *number of partners* variable refers to the number of relationships that the participant reported in the survey. The maximum number of partners a participant could report was five. If the participant had six partners or more, only the most recent five partners would be in the dataset. In our population, only 42 people (2.2%) had five partners in the dataset. In addition, if a respondent had more than five partners and could not report them during the ACASI interview, some of his/her partners may have done so during their own interviews. The relationship may thus have been included in the dataset. Therefore, we believe that the total number of partners was likely not truncated for the vast majority of participants.

We hypothesise that individuals who have relationships with people spanning different birth cohorts may act as ‘bridges’ for HIV to enter other age groups, and thus we wanted a measure that could capture the range in age differences that an individual participant may have. Here we define *bridge width* to be the count of years between the maximum and minimum age difference for an individual. For example, if a woman who was 20 years old at the time of the survey reported two partners: one who was 35 years old and another partner 19 years old at the time of the survey, her *bridge width* would be 16. Finally, the HIV status of participants was determined through rapid HIV testing during LNS data collection^20^,^21^.

### Statistical Analysis

Statistical analysis was performed using R version 3.2.2^22^. Our dataset had missing data for many of the key variables used in the analyses (see Supplementary Tables S1–S4). Based on the specific pattern of missingness we observed, we believe that the data were missing at random (MAR). In order to prevent selection bias associated with using complete cases data in an MAR scenario, we used Multivariate Imputation by Chained Equations (MICE), with a Random Forest (RF) algorithm^23^ to impute missing values^23^,^24^,^25^,^26^ (see Supplementary Note S1).

We described the age-mixing pattern by fitting a generalised linear mixed effects model to regress *age of partner* on *age of participant*, with a random intercept for participant, since we have multi-level data. This mixed effects model captures the average partner age, the between-individual variance, and the within-individual variance. The *between-individual variance* is defined as the population-level variance of the participant-specific average age difference between each participant and their partners. The *within-individual variance* refers to how much the age differences between a participant and his or her partners vary around that participant’s average age difference with his or her partners. The model allowed for heteroskedastic residual variance using a power variance function structure, since preliminary explorations of the data suggested that the variance of the residuals increases with increasing age of the participant.

Next we used generalized linear mixed effects models with a random intercept on participants to determine if *age difference* is associated with relationship characteristics in men and women. We also calculated the median *bridge widths* for men and women among participants who reported more than one partner. Then we used this sub-population of participants and examined the relationship between person-specific characteristics and *bridge width* – our outcome variable. To do this we used negative binomial regression, with the imputed datasets, stratified by gender. Negative binomial regression is useful when the outcome of interest represents overdispersed count data that is bounded by zero. We exponentiated the coefficients produced by the negative binomial model in order to obtain an Expected Bridge Width Ratio (EBWR). In both the *bridge width* and *age difference models* we were only concerned with finding predictors of these age difference constructs using marginal associations, rather than building causal models that adjust for potential confounders.

Finally, we examined the association between our three age difference measures and prevalent HIV using logistic regression to calculate adjusted Odds Ratios (aORs) and 95% confidence intervals (95% CIs). We categorized *mean age difference* and *max age difference* (6 or more years younger/1–5 years younger/0–4 years older/5–9 years older/10 or more years older) while keeping *bridge width* as a linear term in the models. All models were adjusted for *number of partners*, as well as squared and cubic polynomial terms for *age of participant*. The analyses were stratified by gender.

Choosing cut-off points for the categories in our analytical models has a three-fold purpose: 1. It is convention and makes it easy to compare results across studies and settings (some prominent examples include^6^,^13^,^14^), 2. It also allows us to directly compare the men and women in our study population, and 3. It permits us to pool estimates from models repeated on all 50 imputed datasets. However, we also wanted to qualitatively describe the relationship between our age difference measures and the probability of being HIV infected, without forcing a linear or parametric relationship. We suspected the relationship between age difference and HIV was more complex than our parametric analytical models allowed us to observe. Therefore, we randomly selected one of the imputed datasets and constructed Generalised Additive Models (GAMs)^27^ to use as the basis for plots that show the graphical relationship between the age difference measures and the probability of having HIV. These semiparametric models used P-spline smoothers for the age difference measure, and adjust for the same terms as our logistic regression models described above. Unfortunately, the estimates from GAMs cannot be pooled as required for multiply imputed data.

### Ethical Approval

Institutional Review Boards at the University of Malawi College of Medicine and at the University of Pennsylvania approved the LNS. Institutional Review Board approval to conduct this secondary analysis was obtained from the Stellenbosch University Health Research Ethics Committee (IRB0005239). The methods were carried out in accordance with the approved guidelines. Informed consent was obtained from all participants in the LNS. For this secondary analysis no participants were contacted and no new data were collected for this analysis.

## Results

### Age-mixing pattern

**Figure 1:**
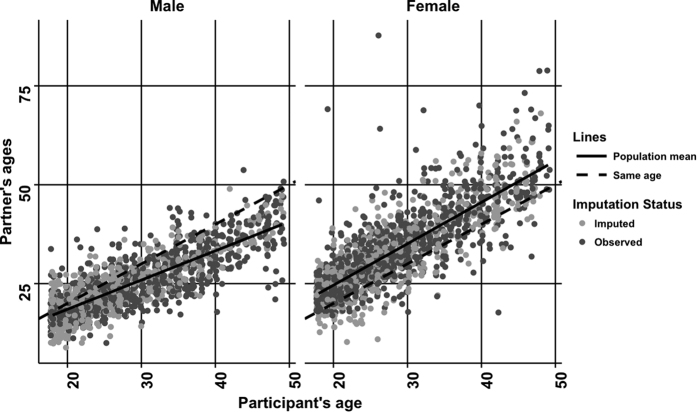
Scatter plot of participant ages versus their partners’ ages. We used a randomly selected dataset from the 50 datasets we imputed to construct the model. The population mean line resulted from the model predictions.

Our survey sample was comprised of 1068 women and 854 men reporting on 1648 and 1688 relationships, respectively. Figure 1 illustrates the age-mixing pattern, with the predictions from the model superimposed. In both genders there was a positive linear relationship between age of participant and partners’ ages. For each year increase in male participant age, the average age difference with his partners increased by 0.26 years. The average age difference between female participants and their partners remained approximately constant around 5 years. In 18 year olds, the between-individual variance in partner ages was slightly higher for women compared to men (1.66 [95% CI: 0.33–8.33] vs. 1.21 [95% CI: 0.63–2.33], respectively). In the same aged participants the within-individual variance was 9.16 (95% CI: 7.69–10.92) for men and 17.05 (95% CI: 13.62–21.35) for women.

### Association between relationship and participant characteristics with age difference

Table 1 shows that there were smaller age differences in relationships where the last sexual episode took place more than a year ago compared to within the last month. This was true for men (β = −0.81, 95% CI: −1.44 to −0.19) and women (β = −1.67, 95% CI: −2.52 to −0.82). In relationships of male participants, the average age difference was 1.56 years smaller with ‘steady partners’ compared to spouses, while in women the age difference was 2.19 years smaller. The other non-spousal partnerships were also associated with smaller age differences. Those that had ever used a condom with their partner also had reduced age differences: almost 1 year less for men and 1.35 years for women. Women in relationships with men living in other villages on Likoma at the time of the relationship had, on average, a 1.21 years (95% CI: −2.1 to −0.31) smaller age difference than those with partners in the same village. Among men who believed that their partner had another partner outside of the relationship, there were, on average, larger age differences (β = 1.02, 95% CI: 0.15–1.89) compared to men who thought their partners never had any other partners while with them. Finally, in both men and women, relationships that were ongoing at the time of the survey had larger age differences than those that had been terminated.

In our sample, 875 participants reported more than one partner in the past three years and had a median bridge width of 4 years (IQR: 1.5–6.5). Specifically for men the median was 3.5 years (IQR: 1.1–5.9) and for women it was 5 years (IQR: 2.5–7.5). Out of all the participants who had more than one partner, only 12 (1.4%) had a bridge width equal to zero, i.e., all their partners had the same age difference. Table 2 illustrates that among men reporting more than one partner, age, marital status, and concurrency were all predictive of larger bridge widths. Older men were more likely to have larger bridge widths. The expected bridge width reported by men increased by 3% for each year increase in age (EBRW: 1.03, 95% CI: 1.01–1.04). Larger bridge widths were also expected among married men (EBWR: 1.38, 95% CI: 1.02–1.87) and those with a concurrent relationship in the past three years (EBWR: 1.43, 95% CI: 1.06–1.92). Among women, the only predictor of larger bridge widths was age (EBWR: 1.02, 95% CI: 0.99–1.05).

### Relationship between age difference measures and HIV status

In the parametric analysis of the association between our age difference measures and HIV status (Table 3), the number of partners a participant had was associated with prevalent HIV in all female models. However, it was only marginally associated with increased likelihood of HIV in male mean and max age difference models. Men who were, on average, six or more years younger than their partners, had five times higher odds of being HIV positive (95% CI: 0.93–31.24) than men who were 0–4 years older than their partners. Regarding women, none of the models demonstrated a relationship between our categorized age difference constructs and HIV status.

**Figure 2:**
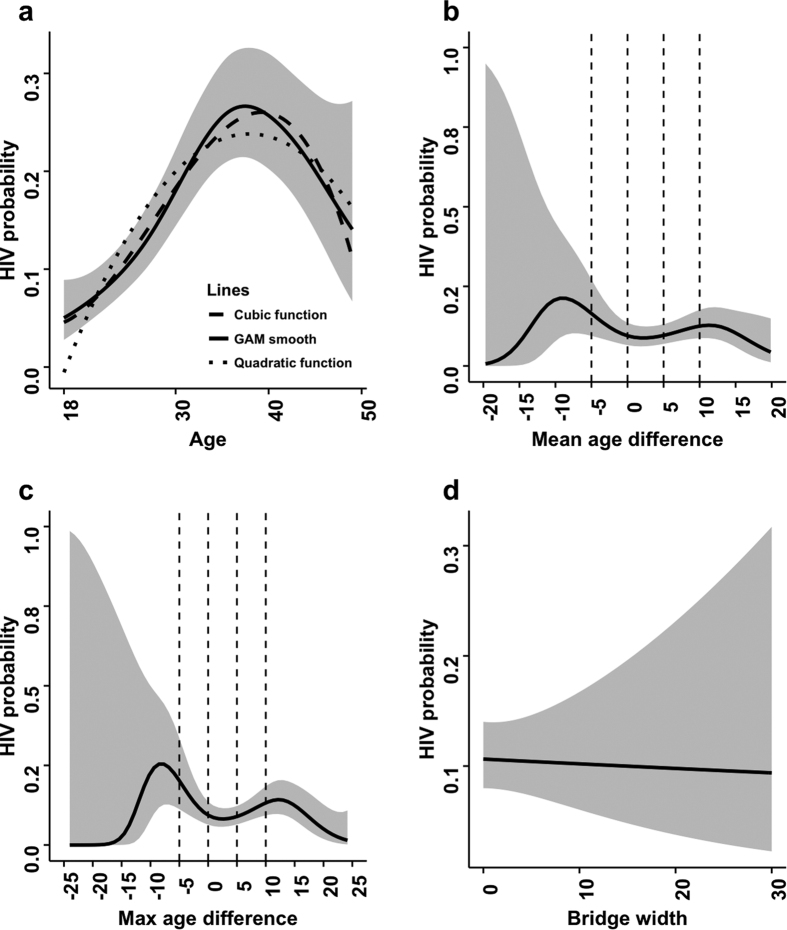
Smoothed plots showing the probability of being HIV-positive among women. The model in panel (a) is univariate and used to demonstrate the functional form that age should take in the regression models. The confidence bands presented result from the GAM. Models in panels (b) to (d) adjust for age, age squared, age cubed, and total number of partners in the past 5 years. For comparison purposes, the chosen cut-points to categorize the age difference measure from the parametric analysis are indicated by vertical dashed lines.

**Figure 3:**
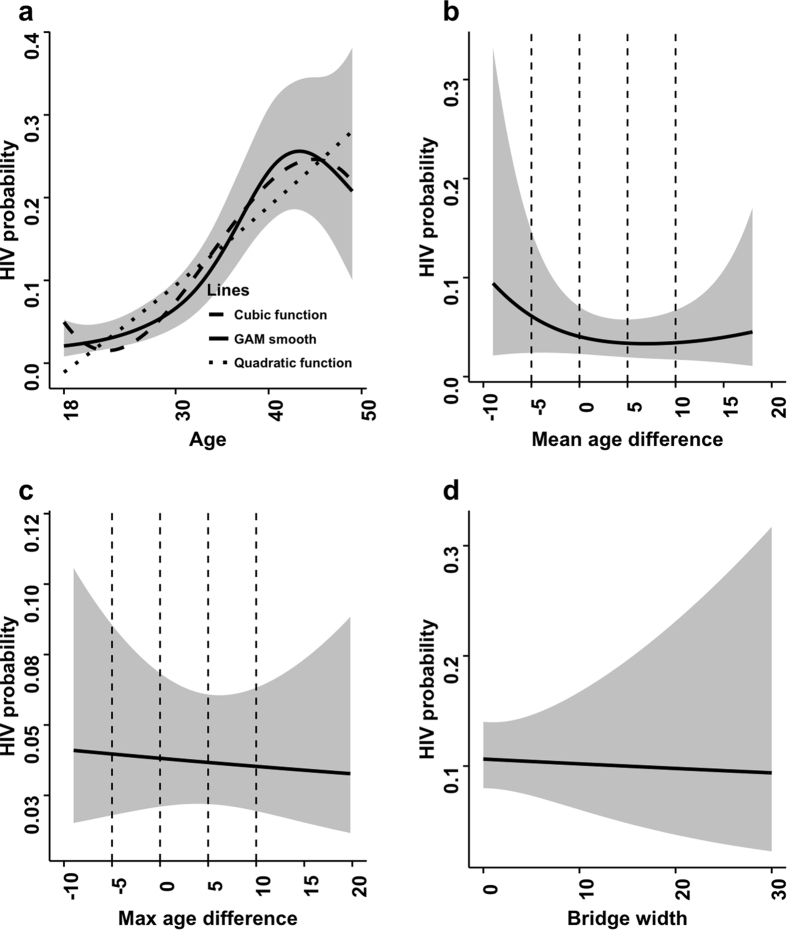
Smoothed plots showing the probability of being HIV-positive among men. The model in panel (a) is univariate and used to demonstrate the functional form that age should take in the regression models. The confidence bands presented result from the GAM. Models in panels (b) to (d) adjust for age, age squared, age cubed, and total number of partners in the past 5 years. For comparison purposes, the chosen cut-points to categorize the age difference measure from the parametric analysis are indicated by vertical dashed lines.

Figures 2 and 3 depict the results of our gender-stratified semiparametric analysis. The relationship between participant age and the probability of being HIV positive appears to most closely resemble a cubic function (Figs 2a and 3a), and is thus the reason why we used that functional form in our regression models. HIV probability increases slowly until about 28 years in women and 33 years in men, and then increases more rapidly until 38 years and 43 years, respectively. Thereafter, the probability starts to decline. Of particular note is the bimodal relationship between mean and max age difference and HIV probability observed in women (Fig. 2b,c). Women who are older than their partners tend to have an increased probability of being HIV positive, but the risk decreases the closer the partner is to her own age. Then the risk for women starts to gradually increase again as their partners become older. When the male partner is 12 or more years older, the risk declines again. A more pronounced, but qualitatively similar pattern is observed for max age difference. When examining the mean age difference plot for men (Fig. 3b), a markedly different relationship is observed. Male participants who were younger than their partners had an increased probability of being HIV positive, and that risk declined as their ages became more similar. Figures 2 and 3 should be interpreted with caution, particularly at extreme values for the age difference measures since the confidence intervals are large due to the sparse number of observations.

## Discussion

Our study quantified the age-mixing pattern among sexually active adults on Likoma Island in Malawi, and explored several ways of analysing the relationship between age difference and prevalent HIV. We considered several age difference measures, as well as parametric and semiparametric regression techniques and discovered that the relationship between HIV and age difference may be more complex than previously described.

We found an age-mixing pattern of increasing average age differences as men get older. For women there is a constant average age difference of approximately five years. This pattern is similar to that observed in a study of adolescents and young adults in Cape Town, South Africa^11^. Additionally, a study that took place in rural KwaZulu-Natal, South Africa also found that age differences in current partners of women who were aged 18–30 years old were approximately four years older. From ages 30–80, the age gaps slowly decreased to almost zero^28^. In our study, we only examined participants 18–49 years old, so we do not know if we would witness a similar decrease if older women had been included in the study. It is possible that in the South African context of extremely high HIV prevalence – where men get tested and treated less often than women – as women become widows in their middle-aged years they begin to take new partners that are closer to their own age.

The findings from our parametric regression analysis that examined the associations between three age difference measures and HIV status are in apparent agreement with recent studies conducted in South Africa. Harling *et al.* did not find evidence that among women 15–30 years old, age differences were associated with increased hazard of HIV acquisition^18^. Likewise, Street *et al.* also could not reject the null hypothesis that women who were in age-disparate relationships would have the same HIV incidence as those in similar-age relationships^14^. However, our results from the semiparametric analysis indicate that this may not be the full story. In particular, women may have some increased risk of being HIV positive if their male partners are older than them by approximately 2–12 years, but then may be slightly protected if their partners are more than 12 years older. The analysis conducted by Street *et al.* produced similar, albeit non-significant, trends: the HIV incidence among women in steady sexual partnerships was 5.78 per 100 person-years with partners who were 0–4 years older, 7.50 with partners 5–9 years older, and 3.67 with partners 10 or more years older^14^. While in our study the associations between mean/max age differences and HIV status in women were not statistically significant, we do believe there is in fact a bimodal association that is meaningful and relevant for HIV transmission dynamics.

Our age-mixing study goes beyond describing mean age differences, and breaks down the variation of age differences into a between-individual and within-individual component. The relatively large within-individual variation in partner ages for young women means that there are opportunities to acquire HIV from men in one age group and then transmit to men in another age group. The potential for transmission between age groups is particularly high because in both men and women in our study, individuals who had larger age differences were more likely to be in spousal relationships, never use a condom during sex, and have had sex in the month prior to the survey. Moreover, men who had a partner whom they thought had a simultaneous relationship, also had larger age differences.

Largely absent from the discourse about how age differences influence HIV epidemiology is how men are affected by age-asymmetric relationships. In our study population, it was not uncommon for men to have had some relationships where the female partner was older than them. Importantly, our study suggests that these men may be at increased risk of having HIV. While not specifically examining age asymmetry and HIV status, a recent study of youth in Eastern Cape, South Africa has also demonstrated that older partner age was associated with curable STIs among boys^29^. Additionally, Gregson *et al.* found that men who chose partners of similar age were more likely to be HIV infected^12^.

One crucial limitation of our study is that the extreme values for the age difference measures in our semiparametric analysis had very large confidence intervals, and therefore, these areas should not be over-interpreted. Secondly, the cross-sectional nature of our study means that we could not determine causality between age difference measures and HIV status. Using complex, semiparametric models requires large sample sizes and so the link between age difference measures and incident HIV could be explored through simulations in a data-driven model world when conducting a large prospective study is not feasible.

We likely also had too few observations in our analysis of the association between bridge widths and HIV status, since calculating a bridge width requires reporting at least two relationships. Less than half of the participants reported more than one. However, we believe that bridge width has the potential to be an important individual-level indicator of variability in partner ages, and should be considered in future studies of age-asymmetry and HIV transmission dynamics, particularly in contexts where individuals are inclined to report more than one partner. In a previous modelling study, it was found that small changes to the variance in the distribution of age differences is enough to increase the basic reproductive number for HIV transmission^4^. The same study also concluded that if the variance in the distribution was large, the mean age difference for the population did not matter much for transmission. We found that men who were married and those who reported a concurrent partner in the past three years were inclined to have larger bridge widths. Moreover, there was also a relationship between older ages and increasing bridge widths. Taking these associations together with the observation that the highest prevalence of HIV among men occurs between the ages of 35 and 45 years implies that there is great HIV transmission potential between different age groups.

Despite these limitations, we believe that our study boasts several strengths. First, we employed two different methods for examining associations between age difference measures and prevalent HIV. The use of GAMs allowed us to build a flexible model that did not impose a specific parametric response function^27^. We also constructed parametric models, which produced no evidence for an association between age difference and HIV status. The benefit of constructing these two types of models, fitting the same data, allowed us to investigate the implication of applying traditional, parametric models to data that require more flexible, nuanced models. Traditionally in analyses of age difference and HIV, if age difference was not treated as a linear term in models, then it was categorized. These arbitrary categories may not adequately capture the true underlying risk patterns^30^. An additional advantage of smooth plots is the ability to visually compare risk among any two or more values of the age difference measures, instead of just comparing to one reference group. For example, we were able to see that women who had a maximum age difference of eight years with their partners had considerably higher risk than those who were at most three years younger than their partners.

Another strength of our study was that the survey data for this secondary analysis was collected using ACASI. ACASI is believed to help elicit more reporting of sensitive behaviours in African contexts^31^,^32^ and to reduce social desirability bias^33^. Thus we believe the reports of relationship characteristics and sexual behaviours in our study were likely more accurate compared to those from surveys where face-to-face interviewing was conducted.

The study of age-asymmetry and HIV transmission dynamics is over two decades old, but the degree to which we understand how choice in partner age influences HIV is still in its infancy. In particular, the role women may play in HIV transmission pathways that connect men from different age groups is still largely unexplored. Our study provides evidence suggesting that the way in which we analyse age-asymmetry may be overly simplistic and require more flexible models to determine the real and complex relationship that age difference, age, and number of partners have to HIV. We believe a crucial step in this line of research is to employ similar analytical techniques to evaluate how different age difference measures – particularly bridge widths – relate to HIV incidence in either large cohorts from different settings or simulation studies modelled after real-world contexts. Though we found that women have large within-individual variation in age difference, we still lack the empirical evidence base to confirm or reject the notion that variation in partner age is a critical driver of individual-level risk of prevalent and incident HIV infection.

## Additional Information

**How to cite this article**: Beauclair, R. *et al.* Age differences between sexual partners, behavioural and demographic correlates, and HIV infection on Likoma Island, Malawi. *Sci. Rep.* **6**, 36121; doi: 10.1038/srep36121 (2016).

**Publisher’s note**: Springer Nature remains neutral with regard to jurisdictional claims in published maps and institutional affiliations.

## Supplementary Material

Supplementary Information

## Figures and Tables

**Table 1:** Association between age difference and other relationship characteristics.

Variables	Age difference Unadjusted β (95% CI)	
	Men	Women
Relationship is ongoing		
No	Ref	Ref
Yes	0.96 (0.42 - 1.50)	1.28 (0.53 - 2.04)
Partner type		
Spouse	Ref	Ref
Steady partner	−1.56 (−2.32 - −0.80)	−2.19 (−3.16 - −1.22)
Infrequent partner	−0.96 (−1.64 - −0.27)	−1.58 (−2.59 - −0.57)
One-night stand	−1.36 (−2.25 - −0.46)	−1.70 (−3.14 - −0.26)
Last sex with partner		
Within last month	Ref	Ref
Within last year	−0.40 (−1.03 – 0.23)	−0.80 (−1.73 - 0.13)
More than a year ago	−0.81 (−1.44 - −0.19)	−1.67 (−2.52 - −0.82)
Had another partner in relationship		
No	Ref	Ref
Yes	−0.20 (−0.77 – 0.36)	−0.48 (−1.40 – 0.45)
Partner had another partner in relationship		
No	Ref	Ref
Yes	1.02 (0.15 - 1.89)	−0.18 (−1.20 - 0.83)
Yes, suspected	0.41 (−0.25 - 1.06)	0.08 (−0.95 - 1.10)
Do not know	0.46 (−0.32 - 1.24)	0.26 (−0.84 - 1.36)
Sex frequency		
Everyday	Ref	Ref
Several times/week	−0.37 (−1.33 - 0.59)	−0.82 (−2.12 - 0.48)
Once/week	−0.57 (−1.54 - 0.41)	−0.93 (−2.24 - 0.39)
Less than once a week	−0.76 (−1.73 - 0.20)	−0.97 (−2.30 - 0.35)
Ever used a condom in relationship		
No	Ref	Ref
Yes	−0.61 (−1.20 - −0.02)	−1.35 (−2.13 - −0.57)
Residence of partner while in the relationship		
Same village on Likoma	Ref	Ref
Other villages of Likoma	−0.31 (−0.94 - 0.31)	−1.21 (−2.10 - −0.31)
In town on Likoma	−0.07 (−1.06 - 0.91)	−0.36 (−1.78 – 1.05)
Mainland Malawi	−0.52 (−1.42 – 0.39)	−0.57 (−1.73 – 0.59)
Chizumulu	0.21 (−1.33 – 1.76)	−0.08 (−2.11 – 1.96)
Mozambique	0.68 (−1.05 – 2.41)	−0.89 (−3.26 – 1.48)

These are pooled estimates from 50 imputed datasets.

CI, Confidence Interval.

**Table 2:** Association between bridge width and person-specific characteristics among participants reporting more than one partner in the previous 3 years.

Variables	Crude EBWR (95% CI)	
	Men	Women
Age	1.03 (1.01 - 1.04)	1.02 (0.99 - 1.05)
Highest level of education		
None or Primary	Ref	Ref
Secondary or Tertiary	0.81 (0.60 - 1.09)	0.80 (0.53 - 1.22)
Religion		
Anglican	Ref	Ref
Other	1.13 (0.78 - 1.65)	0.90 (0.52 - 1.55)
Electrified home		
No	Ref	Ref
Yes	0.80 (0.54 - 1.18)	0.92 (0.50 - 1.69)
Owns own home		
No	Ref	Ref
Yes	1.16 (0.77 - 1.74)	1.44 (0.76 - 2.72)
Marital status		
Never Married	Ref	Ref
Divorced, Widowed, Separated	1.00 (0.44 - 2.26)	1.09 (0.60 - 2.01)
Married	1.38 (1.02 - 1.87)	0.89 (0.57 - 1.38)
Condom use		
Never used a condom with partners	Ref	Ref
Used condoms at some point	0.86 (0.59 - 1.25)	1.18 (0.74 - 1.88)
Non-spousal relationships		
Had a non-spousal partner	Ref	Ref
Only had spousal partners	1.05 (0.73 - 1.50)	0.49 (0.20 - 1.20)
Had a once-off relationship		
No	Ref	Ref
Yes	1.14 (0.83 - 1.55)	0.83 (0.50 - 1.37)
Had a concurrent relationship in past 3 years		
No	Ref	Ref
Yes	1.43 (1.06 - 1.92)	1.01 (0.68 - 1.50)
At least 1 partner definitely had another partner		
No	Ref	Ref
Yes	1.28 (0.93 - 1.75)	1.22 (0.82 - 1.81)
Have a partner that currently resides outside of Likoma		
No	Ref	Ref
Yes	0.78 (0.58 - 1.05)	0.83 (0.55 - 1.26)

These are pooled estimates from 50 imputed datasets.

EBWR, Expected Bridge Width Ratio.

CI, Confidence Interval.

**Table 3:** Association between age difference measures and HIV status.

Variables	Men aOR (95% CI)			Women aOR (95% CI)		
	Mean AD Model	Maximum AD Model	BW Model	Mean AD Model	Maximum AD Model	BW Model
Number of partners	1.28 (0.98 – 1.67)	1.29 (0.98 – 1.69)	1.17 (0.84 – 1.63)	1.51 (1.22 – 1.87)	1.47 (1.19 – 1.82)	1.48 (1.16 – 1.90)
Age of participant	1.01 (0.70 – 1.45)	1.01 (0.70 – 1.45)	1.00 (0.70 – 1.44)	1.04 (0.84 – 1.29)	1.04 (0.83 – 1.29)	1.04 (0.84 – 1.29)
Age of participant squared	1.01 (0.99 – 1.04)	1.02 (0.99 – 1.04)	1.01 (0.99 – 1.04)	1.01 (0.99 – 1.03)	1.01 (0.99 – 1.03)	1.01 (0.99 – 1.03)
Age of participant cubed	1.00 (1.00 – 1.00)	1.00 (1.00 – 1.00)	1.00 (1.00 – 1.00)	1.00 (1.00 – 1.00)	1.00 (1.00 – 1.00)	1.00 (1.00 – 1.00)
Mean age difference						
0–4 years older	Ref	–	–	Ref	–	–
6 or more years younger	5.39 (0.93 – 31.24)	–	–	2.31 (0.60 – 8.98)	–	–
1–5 years younger	0.89 (0.13 – 6.00)	–	–	1.20 (0.55 – 2.64)	–	–
5–9 years older	1.43 (0.68 – 2.98)	–	–	1.04 (0.62 – 1.76)	–	–
10 or more years older	0.80 (0.30 – 2.10)	–	–	1.40 (0.78 – 2.51)	–	–
Maximum age difference						
0–4 years older	–	Ref	–	–	Ref	–
6 or more years younger	–	4.08 (0.40 – 41.16)	–	–	2.51 (0.63 – 9.99)	–
1–5 years younger	–	1.79 (0.36 – 8.83)	–	–	1.53 (0.68 – 3.42)	–
5–9 years older	–	1.58 (0.71 – 3.51)	–	–	1.18 (0.69 – 2.02)	–
10 or more years older	–	1.16 (0.47 – 2.88)	–	–	1.51 (0.86 – 2.65)	–
Bridge width	–	–	1.04 (0.95 – 1.13)	–	–	1.00 (0.94 – 1.06)

These are pooled estimates from 50 imputed datasets.

aOR, adjusted Odds Ratio.

CI, Confidence Interval.

AD, Age Difference.

BW, Bridge Width.

## References

[b1] Anderson R. M., May R. M., Ng T. W. & Rowley J. T. Age-Dependent Choice of Sexual Partners and the Transmission Dynamics of HIV in Sub-Saharan Africa. Philosophical Transactions: Biological Sciences 336, 135–155 (1992). 1353263 10.1098/rstb.1992.0052

[b2] Bershteyn A., Klein D. J. & Eckhoff P. A. Age-dependent partnering and the HIV transmission chain: a microsimulation analysis. Journal of the Royal Society, Interface/the Royal Society 10, 20130613, 10.1098/rsif.2013.0613 (2013). PMC3785829 23985734

[b3] Garnett G. P. & Anderson R. M. Sexually transmitted diseases and sexual behavior: insights from mathematical models. The Journal of infectious diseases. 174 Suppl 2, S150–S161 (1996). 8843245 10.1093/infdis/174.supplement_2.s150

[b4] d’Albis H., Augeraud-Veron E., Djemai E. & Ducrot A. The dispersion of age differences between partners and the asymptotic dynamics of the HIV epidemic. Journal of biological dynamics. 6, 695–717, doi: 10.1080/17513758.2012.688146 (2012). 22873613

[b5] Hallett T. B., Gregson S., Lewis J. J., Lopman B. A. & Garnett G. P. Behaviour change in generalised HIV epidemics: impact of reducing cross-generational sex and delaying age at sexual debut. Sexually transmitted infections. 83 Suppl 1, i50–i54, doi: sti.2006.02360610.1136/sti.2006.023606 (2007). 17314125 10.1136/sti.2006.023606

[b6] Chapman R. *et al.* Do behavioural differences help to explain variations in HIV prevalence in adolescents in sub-Saharan Africa? Tropical Medicine and International Health. 15, 554–566 (2010). 20345559 10.1111/j.1365-3156.2010.02483.x

[b7] Shisana O. *et al.* South African National HIV Prevalence, Incidence and Behaviour Survey, 2012 (HSRC Press, Cape Town, 2014).

[b8] Pettifor A. E. *et al.* Young people’s sexual health in South Africa: HIV prevalence and sexual behaviors from a nationally representative household survey. Aids. 19, 1525–1534, doi: 00002030-200509230-00012 (2005). 16135907 10.1097/01.aids.0000183129.16830.06

[b9] Office N. S. & Macro I. Malawi Demographic and Health Survey 2010 (2011).

[b10] Morrison-Beedy D., Xia Y. & Passmore D. Sexual risk factors for partner age discordance in adolescent girls and their male partners. Journal of clinical nursing. 22, 3289–3299, doi: 10.1111/jocn.12408 (2013). 24580784 PMC3942802

[b11] Beauclair R., Kassanjee R., Temmerman M., Welte A. & Delva W. Age-disparate relationships and implications for STI transmission among young adults in Cape Town, South Africa. The European journal of contraception & reproductive health care: the official journal of the European Society of Contraception. 17, 30–39, doi: 10.3109/13625187.2011.644841 (2012). 22239263

[b12] Gregson S. *et al.* Sexual mixing patterns and sex-differentials in teenage exposure to HIV infection in rural Zimbabwe. Lancet 359, 1896–1903, doi: S0140-6736(02)08780-910.1016/S0140-6736(02)08780-9 (2002). 12057552 10.1016/S0140-6736(02)08780-9

[b13] Maughan-Brown B., Kenyon C. & Lurie M. N. Partner Age Differences and Concurrency in South Africa: Implications for HIV-Infection Risk Among Young Women. AIDS and behavior. 18, 2469–2476, doi: 10.1007/s10461-014-0828-6 (2014). 25047687 PMC4451824

[b14] Street R. A., Reddy T. & Ramjee G. The generational effect on age disparate partnerships and the risk for human immunodeficiency virus and sexually transmitted infections acquisition. International journal of STD & AIDS. 27, 746–752, doi: 10.1177/0956462415592325 (2016). 26138899

[b15] Kraut-Becher J. R. & Aral S. o. Patterns of age mixing and sexually transmitted infections. International journal of STD & AIDS. 17, 378–383 (2006). 16734958 10.1258/095646206777323481

[b16] Kelly R. J. *et al.* Age differences in sexual partners and risk of HIV-1 infection in rural Uganda. Journal of acquired immune deficiency syndromes. 32, 446–451 (2003). 12640205 10.1097/00126334-200304010-00016

[b17] Kenyon C. R., Vu L. & Menten J. & Maughan-Brown, B. Male Circumcision and Sexual Risk Behaviors May Contribute to Considerable Ethnic Disparities in HIV Prevalence in Kenya: An Ecological Analysis. PloS one. 9, e106230, doi: 10.1371/journal.pone.0106230 (2014). 25171060 PMC4149563

[b18] Harling G. *et al.* Do Age-Disparate Relationships Drive HIV Incidence in Young Women? Evidence from a Population Cohort in Rural KwaZulu-Natal, South Africa. Journal of acquired immune deficiency syndromes. 66, 443–451, doi: 10.1097/QAI.0000000000000198 (2014). 24815854 PMC4097949

[b19] Harling G., Newell M. L., Tanser F. & Barnighausen T. Partner Age-Disparity and HIV Incidence Risk for Older Women in Rural South Africa. AIDS and behavior. 19, 1317–1326, doi: 10.1007/s10461-014-0952-3 (2015). 25670473 PMC4506232

[b20] Helleringer S., Mkandawire J., Kalilani-Phiri L. & Kohler H. P. Cohort Profile: The Likoma Network Study (LNS). International journal of epidemiology. 43, 545–557, doi: 10.1093/ije/dyt001 (2014). 23543589 PMC3997370

[b21] Helleringer S., Kohler H. P., Chimbiri A., Chatonda P. & Mkandawire J. The Likoma Network Study: Context, data collection, and initial results. Demographic research. 21, 427–468, doi: 10.4054/DemRes.2009.21.15 (2009). 20179777 PMC2825888

[b22] R: A language and environment for statistical computing. (R Foundation for Statistical Computing, Vienna, Austria, 2014).

[b23] Shah A. D., Bartlett J. D., Carpenter J., Nicholas O. & Hemingway H. Comparison of Random Forest and Parametric Imputation Models for Imputing Missing Data Using MICE: A CALIBER Study. American journal of epidemiology. 179, 764–774 (2014). 24589914 10.1093/aje/kwt312 PMC3939843

[b24] Penone C. *et al.* Imputation of missing data in life-history trait datasets: which approach performs best? Methods in Ecology and Evolution. 5, 961–970 (2014).

[b25] Stekhoven D. J. & Buhlmann P. MissForest - nonparametric missing value imputation for mixed-type data. Bioinformatics. 28, 112–118 (2012). 22039212 10.1093/bioinformatics/btr597

[b26] van Buuren S. & Groothuis-Oudshoorn K. MICE: Multivariate Imputation by Chained Equations in R. Journal of statistical software. 45, 1–67 (2011).

[b27] Shiboski S. C. Generalized Additive Models for Current Status Data. Lifetime data analysis. 4, 29–50 (1998). 9567054 10.1023/a:1009652024999

[b28] Ott M. Q., Barnighausen T., Tanser F., Lurie M. & Newell M. L. Age-gaps in sexual partnerships: seeing beyond ‘sugar daddies’. Aids. 25, 861–869 (2011). 21358377 10.1097/QAD.0b013e32834344c9 PMC3117250

[b29] O’Leary A. *et al.* Associations between Psychosocial Factors and Incidence of Sexually Transmitted Disease Among South African Adolescents. Sexually transmitted diseases. 42, 135–139 (2015). 25668645 10.1097/OLQ.0000000000000247 PMC4351752

[b30] Bennette C. & Vickers A. Against quantiles: categorization of continuous variables in epidemiologic research, and its discontents. BMC medical research methodology. 12, 21, doi: 10.1186/1471-2288-12-21 (2012). 22375553 PMC3353173

[b31] Dolezal C. *et al.* A comparison of audio computer-assisted self-interviews to face-to-face interviews of sexual behavior among perinatally HIV-exposed youth. Archives of sexual behavior. 41, 401–410, doi: 10.1007/s10508-011-9769-6 (2012). 21604065 PMC3621976

[b32] Hewett P., Mensch B. & Erulkar A. Consistency in the reporting of sexual behavior by adolescent girls in Kenya: a comparison of interviewing methods. Sexually Transmitted Infections 80, ii43–ii48 (2004). 15572639 10.1136/sti.2004.013250 PMC1765856

[b33] Beauclair R. *et al.* Evaluating Audio Computer Assisted Self-Interviews in Urban South African Communities: Evidence for Good Suitability and Reduced Social Desirability Bias of a Cross-sectional Survey on Sexual Behaviour. BMC medical research methodology. 13, doi: 10.1186/1471-2288-13-11 (2013). PMC3568408 23368888

