# Supplementary material for: Age differences between sexual partners, behavioural and demographic correlates, and HIV infection on Likoma Island, Malawi
Source: Sci Rep. 2016 Nov 2;6:36121. doi: 10.1038/srep36121 (PMC5090960; doi:10.1038/srep36121)
Supplement: Supplementary Information [file srep36121-s1.doc]

**Supplementary information for the manuscript entitled:**

***Age differences between sexual partners, behavioural and demographic correlates, and HIV infection on Likoma Island, Malawi***

Roxanne Beauclair ^*, 1,2^, Stéphane Helleringer ^3^, Niel Hens ^4,5,6^, Wim Delva ^1,2,4,7^

1 International Centre for Reproductive Health, Ghent University, Gent, Belgium

2 The South African Department of Science and Technology / National Research Foundation (DST/NRF) Centre of Excellence in Epidemiological Modelling and Analysis (SACEMA), Stellenbosch University, Stellenbosch, South Africa

3 Bloomberg School of Public Health, Johns Hopkins University, Baltimore, USA

4 Center for Statistics, Hasselt University, Hasselt, Belgium

5 Centre for Health Economic Research and Modelling Infectious Diseases, Vaccine and Infectious Disease Institute, University of Antwerp, Wilrijk, Belgium

6 Epidemiology and Social Medicine, University of Antwerp, Wilrijk, Belgium

7 Rega Institute for Medical Research, KU Leuven, Leuven, Belgium

Supplementary Table S1. Comparison of participant characteristics by HIV missing status. All continuous values are reported with median and inter-quartile range, Med (IQR), while categories are reported in percentages, n (%).

|  | **HIV status missing** | |
| --- | --- | --- |
| **Variables** | **No**  **n = 1,474** | **Yes**  **n = 448** |
| Age | 27.0 (22.0 - 34.0) | 29.5 (23.0 - 36.0) |
| Gender | | |
| Male | 612 (41.5%) | 242 (54.0%) |
| Female | 862 (58.5%) | 206 (46.0%) |
| Highest level of education | | |
| None or Primary | 863 (58.5%) | 238 (53.1%) |
| Secondary or Tertiary | 561 (38.1%) | 195 (43.5%) |
| Missing | 50 (3.4%) | 15 (3.3%) |
| Religion | | |
| Anglican | 1,182 (80.2%) | 330 (73.7%) |
| Other | 263 (17.8%) | 105 (23.4%) |
| Missing | 29 (2.0%) | 13 (2.9%) |
| Electrified home | | |
| No | 1,204 (81.7%) | 348 (77.7%) |
| Yes | 265 (18.0%) | 97 (21.7%) |
| Missing | 5 (0.3%) | 3 (0.7%) |
| Owns own home | | |
| No | 201 (13.6%) | 91 (20.3%) |
| Yes | 1,268 (86.0%) | 354 (79.0%) |
| Missing | 5 (0.3%) | 3 (0.7%) |
| Marital status | | |
| Never Married | 490 (33.2%) | 132 (29.5%) |
| Divorced, Widowed, Separated | 145 (9.8%) | 38 (8.5%) |
| Married | 803 (54.5%) | 264 (58.9%) |
| Missing | 36 (2.4%) | 14 (3.1%) |
| Number of partners | 1.0 (1.0 - 2.0) | 1.0 (1.0 - 2.0) |

Supplementary Table S2. Comparison of participant characteristics for those with any missing partner age versus those with all known partner ages. All continuous values are reported with median and inter-quartile range, Med (IQR), while categories are reported in percentages, n (%).

|  | **Partner age missing** | |
| --- | --- | --- |
| **Variables** | **A partner age is missing**  **n = 896** | **No ages missing**  **n = 1,026** |
| Age | 25.0 (21.0 – 32.0) | 29.0 (23.0 – 36.0) |
| Gender | | |
| Male | 445 (49.7%) | 409 (39.9%) |
| Female | 451 (50.3%) | 617 (60.1%) |
| Highest level of education | | |
| None or Primary | 472 (52.7%) | 629 (61.3%) |
| Secondary or Tertiary | 394 (44.0%) | 362 (35.3%) |
| Missing | 30 (3.3%) | 35 (3.4%) |
| Religion | | |
| Anglican | 716 (79.9%) | 796 (77.6%) |
| Other | 167 (18.6%) | 201 (19.6%) |
| Missing | 13 (1.5%) | 29 (2.8%) |
| Electrified home | | |
| No | 735 (82.0%) | 817 (79.6%) |
| Yes | 158 (17.6%) | 204 (19.9%) |
| Missing | 3 (0.3%) | 5 (0.5%) |
| Owns own home | | |
| No | 132 (14.7%) | 160 (15.6%) |
| Yes | 761 (84.9%) | 861 (83.9%) |
| Missing | 3 (0.3%) | 5 (0.5%) |
| Marital status | | |
| Never Married | 414 (46.2%) | 208 (20.3%) |
| Divorced, Widowed, Separated | 127 (14.2%) | 56 (5.5%) |
| Married | 334 (37.3%) | 733 (71.4%) |
| Missing | 21 (2.3%) | 29 (2.8%) |
| Number of partners | 2.0 (2.0 – 3.0) | 1.0 (1.0 – 1.0) |

Supplementary Table S3. Comparison of relationship characteristics by participants’ HIV missing status. All continuous values are reported with median and inter-quartile range, Med (IQR), while categories are reported in percentages, n (%).

|  | **HIV status missing** | |
| --- | --- | --- |
| **Variables** | **No**  **n = 2,601** | **Yes**  **n = 735** |
| Partner is alive | | |
| Alive | 1,456 (56.0%) | 395 (53.7%) |
| Dead | 145 (5.6%) | 44 (6.0%) |
| Do not know | 8 (0.3%) | 0 (0.0%) |
| Missing | 992 (38.1%) | 296 (40.3%) |
| Relationship is ongoing | | |
| No | 1,608 (61.8%) | 436 (59.3%) |
| Yes | 981 (37.7%) | 292 (39.7%) |
| Missing | 12 (0.5%) | 7 (1.0%) |
| Partner type | | |
| Spouse | 1,025 (39.4%) | 311 (42.3%) |
| Steady partner | 583 (22.4%) | 152 (20.7%) |
| Infrequent partner | 722 (27.8%) | 175 (23.8%) |
| One-night stand | 260 (10.0%) | 93 (12.7%) |
| Missing | 11 (0.4%) | 4 (0.5%) |
| Last sex with partner | | |
| Within last month | 951 (36.6%) | 323 (43.9%) |
| Within last year | 608 (23.4%) | 174 (23.7%) |
| More than a year ago | 1,005 (38.6%) | 223 (30.3%) |
| Missing | 37 (1.4%) | 15 (2.0%) |
| Had another partner in relationship | | |
| No | 2,004 (77.0%) | 557 (75.8%) |
| Yes | 584 (22.5%) | 173 (23.5%) |
| Missing | 13 (0.5%) | 5 (0.7%) |
| Partner had another partner in relationship | | |
| No | 713 (27.4%) | 210 (28.6%) |
| Yes | 569 (21.9%) | 152 (20.7%) |
| Yes, suspected | 852 (32.8%) | 244 (33.2%) |
| Do not know | 425 (16.3%) | 116 (15.8%) |
| Missing | 42 (1.6%) | 13 (1.8%) |
| Sex frequency | | |
| Everyday | 227 (8.7%) | 76 (10.3%) |
| Several times/week | 763 (29.3%) | 224 (30.5%) |
| Once/week | 752 (28.9%) | 179 (24.4%) |
| Less than once a week | 815 (31.3%) | 245 (33.3%) |
| Missing | 44 (1.7%) | 11 (1.5%) |
| Ever used a condom in relationship | | |
| No | 956 (36.8%) | 252 (34.3%) |
| Yes | 1,500 (57.7%) | 373 (50.7%) |
| Missing | 145 (5.6%) | 110 (15.0%) |
| Residence of partner while in the relationship | | |
| Same village on Likoma | 1,166 (44.8%) | 298 (40.5%) |
| Other villages of Likoma | 614 (23.6%) | 155 (21.1%) |
| In town on Likoma | 262 (10.1%) | 67 (9.1%) |
| Mainland Malawi | 377 (14.5%) | 157 (21.4%) |
| Chizumulu | 91 (3.5%) | 24 (3.3%) |
| Mozambique | 73 (2.8%) | 24 (3.3%) |
| Missing | 18 (0.7%) | 10 (1.4%) |

Supplementary Table S4. Comparison of variables for those with missing partner age versus observed partner age. All continuous values are reported with median and inter-quartile range, Med (IQR), while categories are reported in percentages, n (%).

|  | **Partner age missing** | |
| --- | --- | --- |
| **Variables** | **No**  **n = 1,997** | **Yes**  **n = 1,339** |
| Partner is alive | | |
| Alive | 883 (44.2%) | 968 (72.3%) |
| Dead | 51 (2.6%) | 138 (10.3%) |
| Do not know | 2 (0.1%) | 6 (0.4%) |
| Missing | 1,061 (53.1%) | 227 (17.0%) |
| Relationship is ongoing | | |
| No | 929 (46.5%) | 1,115 (83.3%) |
| Yes | 1,055 (52.8%) | 218 (16.3%) |
| Missing | 13 (0.7%) | 6 (0.4%) |
| Partner type | | |
| Spouse | 1,081 (54.1%) | 255 (19.0%) |
| Steady partner | 384 (19.2%) | 351 (26.2%) |
| Infrequent partner | 392 (19.6%) | 505 (37.7%) |
| One-night stand | 131 (6.6%) | 222 (16.6%) |
| Missing | 9 (0.5%) | 6 (0.4%) |
| Last sex with partner | | |
| Within last month | 969 (48.5%) | 305 (22.8%) |
| Within last year | 423 (21.2%) | 359 (26.8%) |
| More than a year ago | 573 (28.7%) | 655 (48.9%) |
| Missing | 32 (1.6%) | 20 (1.5%) |
| Had another partner in relationship | | |
| No | 1,534 (76.8%) | 1,027 (76.7%) |
| Yes | 455 (22.8%) | 302 (22.6%) |
| Missing | 8 (0.4%) | 10 (0.7%) |
| Partner had another partner in relationship | | |
| No | 538 (26.9%) | 385 (28.8%) |
| Yes | 422 (21.1%) | 299 (22.3%) |
| Yes, suspected | 630 (31.5%) | 466 (34.8%) |
| Do not know | 385 (19.3%) | 156 (11.7%) |
| Missing | 22 (1.1%) | 33 (2.5%) |
| Sex frequency | | |
| Everyday | 212 (10.6%) | 91 (6.8%) |
| Several times/week | 647 (32.4%) | 340 (25.4%) |
| Once/week | 559 (28.0%) | 372 (27.8%) |
| Less than once a week | 544 (27.2%) | 516 (38.5%) |
| Missing | 35 (1.8%) | 20 (1.5%) |
| Ever used a condom in relationship | | |
| No | 748 (37.5%) | 460 (34.4%) |
| Yes | 1,085 (54.3%) | 788 (58.8%) |
| Missing | 164 (8.2%) | 91 (6.8%) |
| Residence of partner while in the relationship | | |
| Same village on Likoma | 1,140 (57.1%) | 324 (24.2%) |
| Other villages of Likoma | 495 (24.8%) | 274 (20.5%) |
| In town on Likoma | 153 (7.7%) | 176 (13.1%) |
| Mainland Malawi | 128 (6.4%) | 406 (30.3%) |
| Chizumulu | 43 (2.2%) | 72 (5.4%) |
| Mozambique | 23 (1.2%) | 74 (5.5%) |
| Missing | 15 (0.8%) | 13 (1.0%) |

Supplementary Note S1. Rationale for choice in imputation methods

Multivariate Imputation by Chained Equation (MICE) methods create multiple datasets using other variables in the original dataset to predict missing values. Analysts can then use the same regression models on each dataset and produce pooled estimates, which take into account uncertainty in the imputations [1] and lead to larger confidence intervals. In order to reduce simulation error, that may result from imputing too few datasets we imputed 50 datasets [1]. The main advantage of using the Random Forest (RF) algorithm with MICE imputation is that the algorithm makes few assumptions about the data and therefore, it is ideal for imputing datasets for which there are non-linearities and interactions between key variables [2-4].

**References**

1. van Buuren S, Groothuis-Oudshoorn K. MICE: Multivariate Imputation by Chained Equations in R. Journal of statistical software. 2011;45(3):1-67.

2. Penone C, Davidson AD, Shoemaker KT, DiMarco M, Rondinini C, Brooks TM, et al. Imputation of missing data in life-history trait datasets: which approach performs best? Methods in Ecology and Evolution. 2014;5(9):961-70.

3. Shah AD, Bartlett JD, Carpenter J, Nicholas O, Hemingway H. Comparison of Random Forest and Parametric Imputation Models for Imputing Missing Data Using MICE: A CALIBER Study. American journal of epidemiology. 2014;179(6):764-74.

4. Stekhoven DJ, Buhlmann P. MissForest - nonparametric missing value imputation for mixed-type data. Bioinformatics. 2012;28(1):112-8.
